# Radiological findings of complications after lung transplantation

**DOI:** 10.1007/s13244-018-0647-9

**Published:** 2018-08-15

**Authors:** Céline Habre, Paola M. Soccal, Frédéric Triponez, John-David Aubert, Thorsten Krueger, Steve P. Martin, Joanna Gariani, Jean-Claude Pache, Frédéric Lador, Xavier Montet, Anne-Lise Hachulla

**Affiliations:** 1 Division of Radiology, University Hospitals of Geneva, Rue Gabrielle-Perret-Gentil 4, Geneva, Switzerland; 2 Department of Pneumology, University Hospitals of Geneva, Geneva, Switzerland; 3 Pulmonary Hypertension Program, University Hospitals of Geneva, Geneva, Switzerland; 4 Faculty of Medicine of Geneva, Geneva, Switzerland; 5 Department of Surgery, University Hospitals of Geneva, Geneva, Switzerland; 6 Department of Pneumology, Lausanne University Hospital, Lausanne, Switzerland; 7 University of Lausanne, Lausanne, Switzerland; 8 Department of Surgery, Lausanne University Hospital, Lausanne, Switzerland; 9 Department of Pathology, University Hospitals of Geneva, Geneva, Switzerland

**Keywords:** Lung transplant complications, Radiological findings

## Abstract

**Abstract:**

Complications following lung transplantation may impede allograft function and threaten patient survival. The five main complications after lung transplantation are primary graft dysfunction, post-surgical complications, alloimmune responses, infections, and malignancy. *Primary graft dysfunction*, a transient ischemic/reperfusion injury, appears as a pulmonary edema in almost every patient during the first three days post-surgery. *Post-surgical dysfunction* could be depicted on computed tomography (CT), such as bronchial anastomosis dehiscence, bronchial stenosis and bronchomalacia, pulmonary artery stenosis, and size mismatch. *Alloimmune responses* represent acute rejection or chronic lung allograft dysfunction (CLAD). CLAD has three different forms (bronchiolitis obliterans syndrome, restrictive allograft syndrome, acute fibrinoid organizing pneumonia) that could be differentiated on CT. *Infections* are different depending on their time of occurrence. The first post-operative month is mostly associated with bacterial and fungal pathogens. From the second to sixth months, viral pneumonias and fungal and parasitic opportunistic infections are more frequent. Different patterns according to the type of infection exist on CT. *Malignancy* should be depicted and corresponded principally to post-transplantation lymphoproliferative disease (PTLD). In this review, we describe specific CT signs of these five main lung transplantation complications and their time of occurrence to improve diagnosis, follow-up, medical management, and to correlate these findings with pathology results.

**Key Points:**

• *The five main complications are primary graft dysfunction, surgical, alloimmune, infectious, and malignancy complications*.

• *CT identifies anomalies in the setting of unspecific symptoms of lung transplantation complications*.

• *Knowledge of the specific CT signs can allow a prompt diagnosis*.

• *CT signs maximize the yield of bronchoscopy, transbronchial biopsy, and bronchoalveolar lavage*.

• *Radiopathological correlation helps to understand CT signs after lung transplantation complications*.

## Introduction

Complications following transplantation may impede allograft function and threaten patient survival. Five principal complications have been described: primary graft dysfunction (PGD), post-surgical complications, alloimmune responses, infections, and malignancy [1, 2].

Improvement in surgical procedures, in particular bronchial anastomosis techniques and the endoscopic management of stenosis or leakage, has contributed to reduce airway complications [3, 4]. Furthermore, systematic infection prophylaxis and a trend for virus serology matching have significantly reduced post-operative morbidity and improved long-term survival with reduced chronic allograft dysfunction [5].

Nevertheless, these advances are currently unable to completely prevent these complications and clinical follow-up is required with regular lung function assessments, bronchoscopy exams, and computed tomography (CT) scans.

Chest X-ray is performed systematically every year or whenever clinical symptoms occur. Chest CT is a key tool in active follow-up or when chest X-ray is abnormal by allowing early identification and diagnostic clues in the setting of unspecific acute respiratory symptoms, regardless of their origin. Knowledge of the specific CT signs can improve medical management by allowing a prompt diagnosis, by guiding bronchoscopic procedures for bronchoalveolar lavage and transbronchial biopsy. Likewise, the decline of lung function may not always be discriminative between infection or rejection, either acute or chronic, and further investigation by chest CT may help clinicians in establishing a diagnosis before more invasive bronchoscopic procedures.

**Fig. 1:**
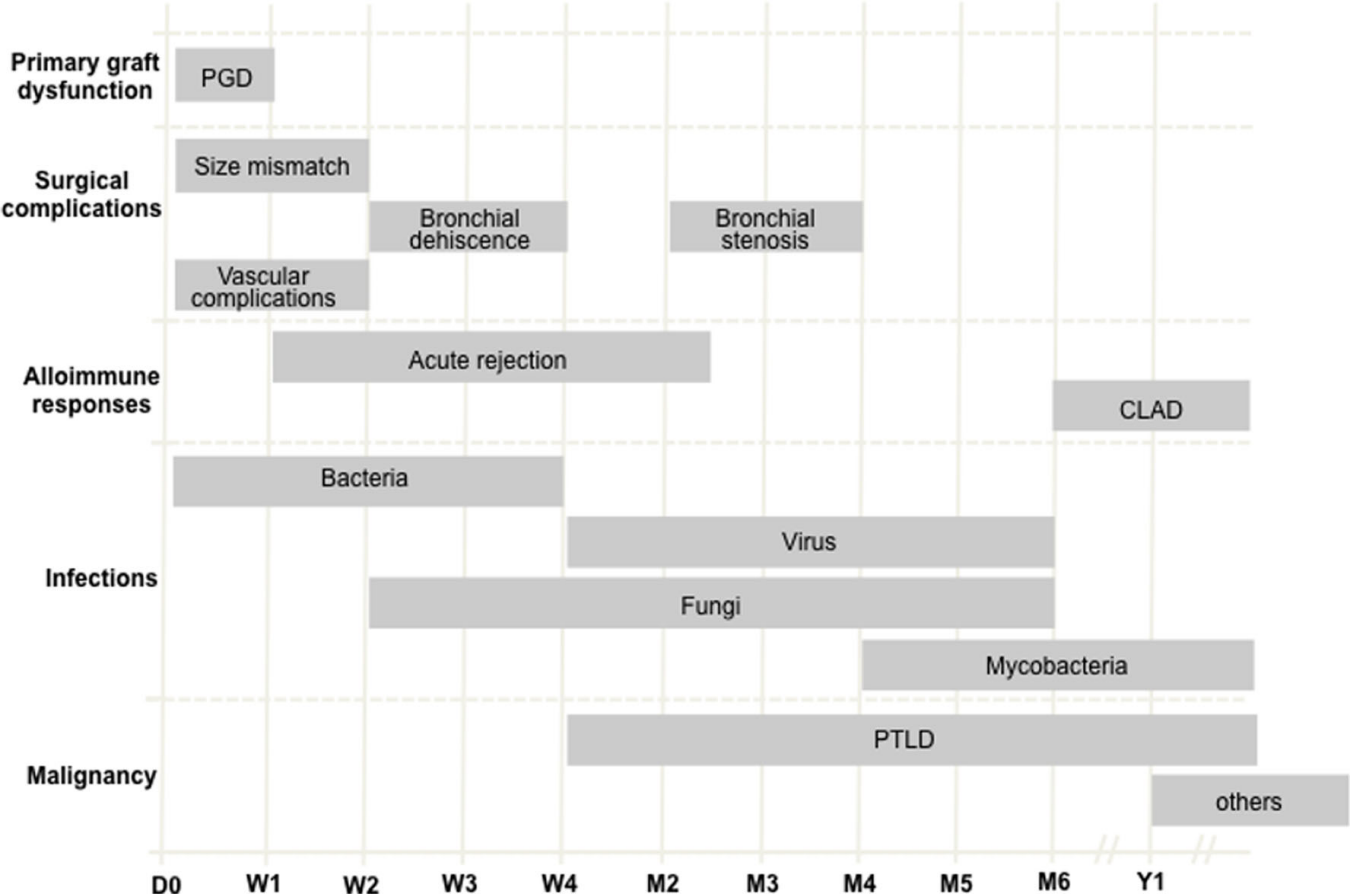
Onset of complications following lung transplantation. Adapted from Ng et al. 2009 [4]. CLAD: chronic lung allograft dysfunction; PGD: primary graft dysfunction; PTLD: post-transplantation lymphoproliferative disease; D: day; W: week; M: month; Y: year

Complications after lung transplantation vary depending on the delay of their occurrence. Figure 1 summarizes the mean time of occurrence and the greatest incidence of complications depending on the time after the procedure and according to the literature [4, 6, 7].

In this review, we describe specific CT signs of these five main lung transplantation complications and their time of occurrence to improve diagnosis, follow-up, medical management, guide bronchoscopic procedures, and to correlate these findings with pathology results.

## Primary graft dysfunction

The first lung transplantation complication to occur is *primary graft dysfunction*. This corresponds to a transient ischemic/reperfusion injury that appears as a pulmonary edema in almost every patient during the first three days post-surgery [8].

**Fig. 2:**
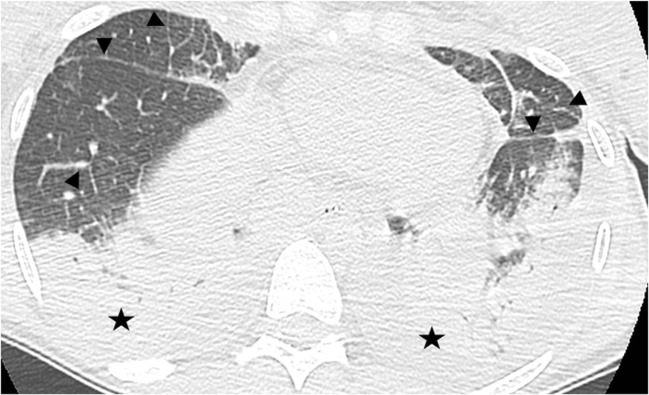
Primary graft dysfunction. Septal, scissural, and peribronchovascular thickening (*arrowheads*) and pleural effusions (*stars*)

PGD is clinically assessed and graded according to the ratio of arterial oxygen pressure to inspired oxygen concentration (PaO_2_/FiO_2_) and presence of lung allograft consolidations on the chest radiograph [4]. Ischemia reperfusion injury should be considered after the exclusion of an infectious or cardiogenic etiology. CT signs are pleural or scissural effusions, septal and peribronchovascular thickening, perihilar consolidations, without cardiomegaly, and sparing of the native lung in cases of single lung transplantation (Fig. 2) [7]. It is interesting to note that exudative and hemorrhagic pleural effusions are a normal finding early after thoracic surgery that may persist until the second month.

## Post-surgical dysfunction

Four different post-surgical complications have been described: bronchial anastomosis dehiscence, bronchial stenosis and bronchomalacia, pulmonary artery stenosis, and size mismatch, as listed in Table 1.

**Table 1:** Computed tomography (CT) findings of post-surgical complications

	Bronchial anastomosis dehiscence	Bronchial stenosis	Bronchomalacia	Pulmonary artery stenosis	Size mismatch
Time of occurrence	2–4 weeks	2–4 months	2–4 months	0–2 weeks	0–2 weeks
Clinical keys	Dyspnea and recurrent infections	Dyspnea Recurrent post-obstructive infections	Dyspnea Recurrent post-obstructive infections	Dyspnea and chest pain	Dyspnea
CT signs	Focal parietal defect Pneumomediastinum Pneumothorax Subcutaneous emphysema	Fixed reduction of bronchial lumen diameter	Dynamic collapse of the bronchus on expiratory acquisition	Reduced caliber Homolateral hypoperfusion Dilatation of main PA and right cavities	Allograft atelectasis

**Fig. 3:**
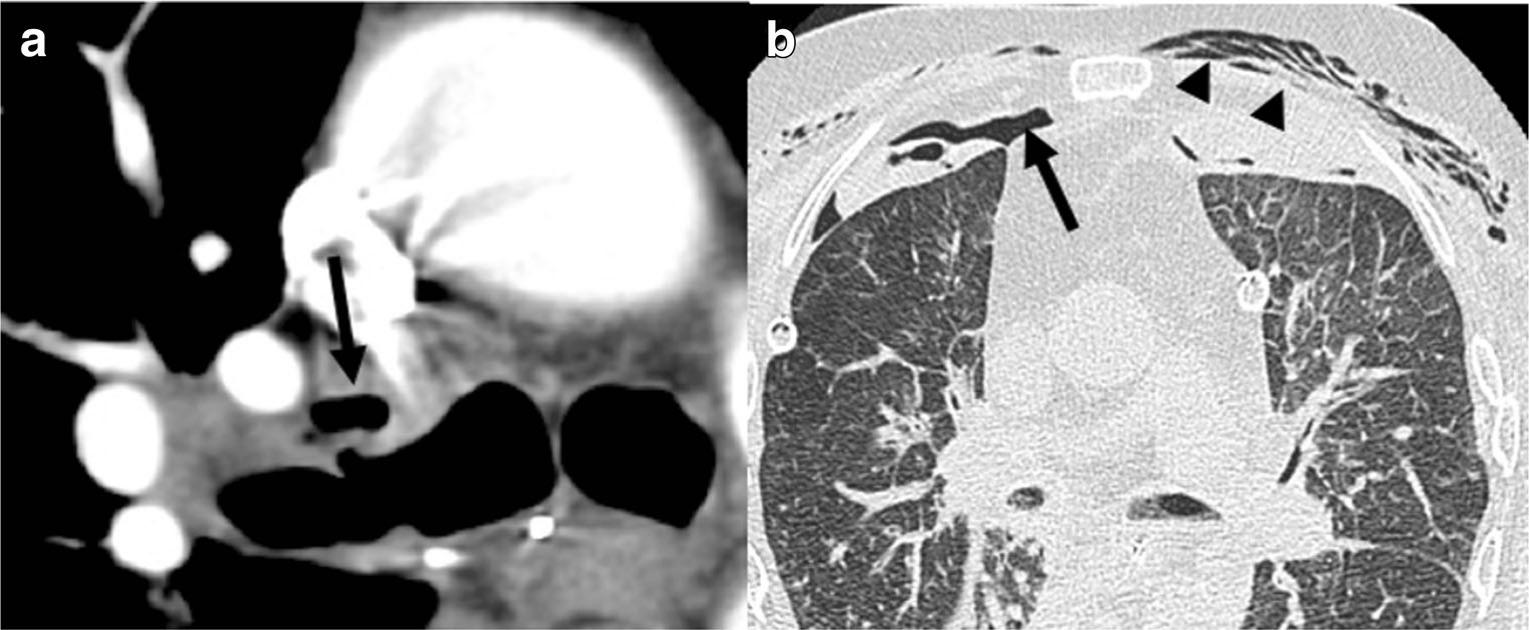
Bronchial anastomosis dehiscence. Air collection anterior to the right bronchial suture (*arrow*) (**a**). Indirect signs include persistent pneumothorax (*arrow*) and new subcutaneous emphysema (*arrowheads*) (**b**)

Bronchial dehiscence of the anastomosis results mainly from bronchial ischemia because of the absence of reanastomosis of bronchial arteries [9]. It may appear as a focal parietal defect or may be suggested indirectly by pneumomediastinum, pneumothorax, or subcutaneous emphysema 2 weeks after surgery (Fig. 3) [10].

**Fig. 4:**
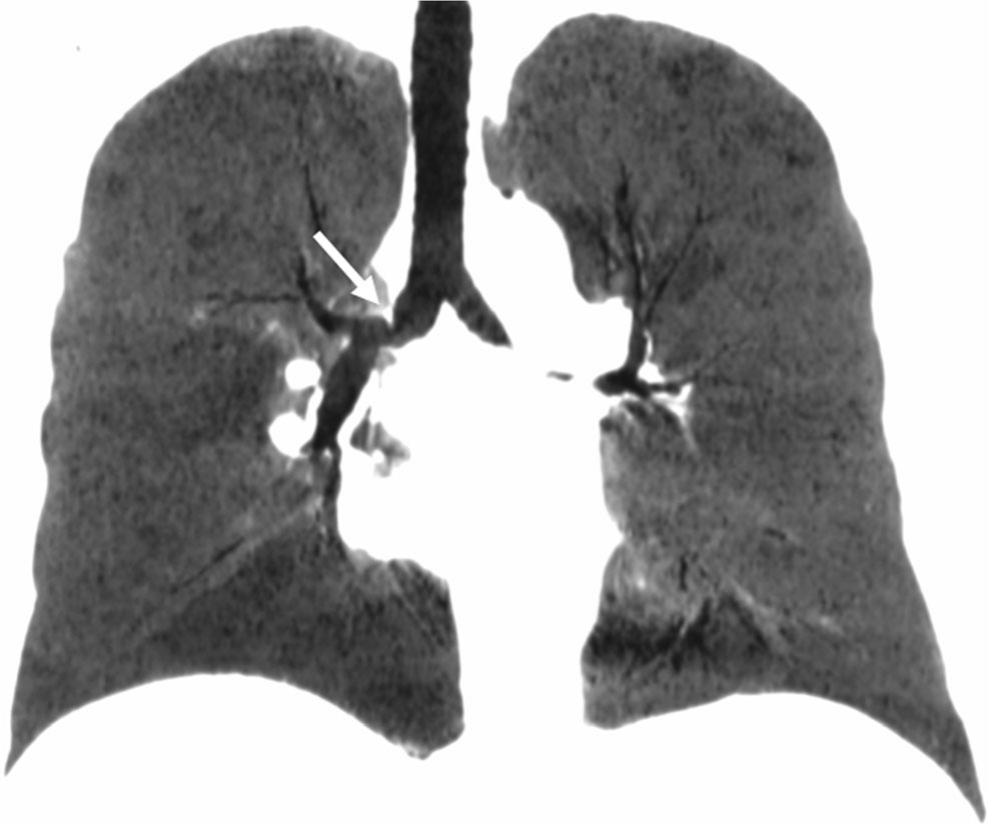
Bronchial stenosis. Dyspnea and recurrent infections at 6 months after lung transplantation. Narrowing of the anastomosis of the right main bronchus with minimal intensity projection (*arrow*)

Afterwards, lesions of bronchial healing from ischemia may also occur, either as bronchial anastomosis stenosis or bronchomalacia [7]. Bronchial stenosis is a fixed reduction of the bronchial lumen diameter and bronchomalacia appears as a dynamic collapse of the bronchus on expiratory acquisition. Both can cause recurrent post-obstructive infections of the involved ventilated regions [4, 5, 7] (Fig. 4).

**Fig. 5:**
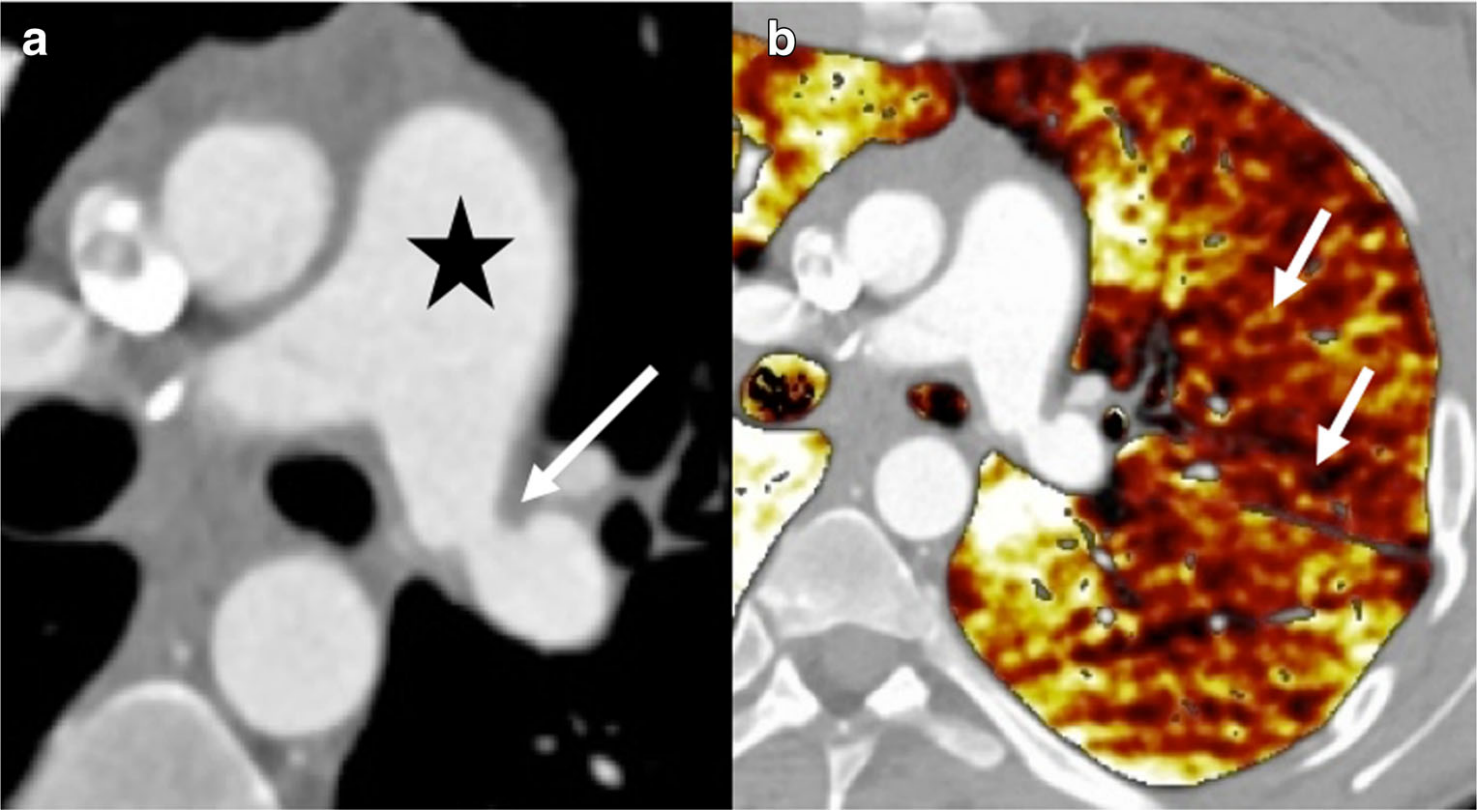
Pulmonary artery stenosis. Dyspnea and chest pain 3 months after surgery. Stenosis of the left PA (*arrow*) responsible for dilatation of the main PA (*star*) (**a**) and, as consequences, a wide hypoperfusion of the left lung in the pale yellow color area on the perfusion map (*arrows*) (**b**)

Pulmonary artery stenosis could be depicted by a significant difference of the pulmonary arterial diameter between donor and receiver, responsible for ipsilateral pulmonary hypoperfusion depicted on the iodine cartography perfusion map with dual-energy CT (Fig. 5) [11]. Dilatation of the main pulmonary artery and right heart cavities have been described.

**Fig. 6:**
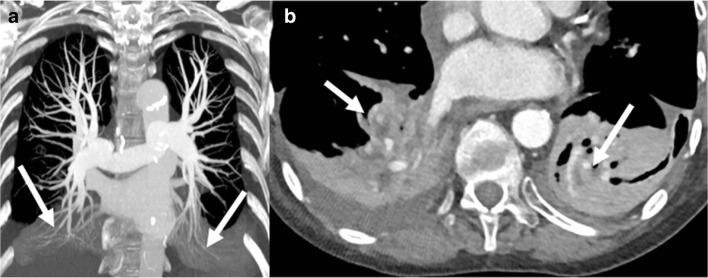
Donor–recipient size mismatch. Persisting dyspnea. Atelectasis of lower lobes (*arrows* in **a**) with comet-tail sign (*arrows* in **b**)

Size mismatch between the donor lung and the recipient thoracic cage can cause areas of atelectasis, or even complete collapse of the allograft in extreme cases (Fig. 6).

## Alloimmune responses

Lung-transplanted patients are at particular risk for alloimmune responses. Acute rejection and chronic lung allograft dysfunction (CLAD) have been described and Table 2 summarizes the alloimmune response findings on CT.

**Table 2:** Specific CT pattern with alloimmune complications

Alloimmune responses	Acute rejection	BOS	RAS	AFOP
Time of occurrence	1–12 weeks	6–18 months	6–18 months	6–18 months
Clinical keys	Acute dyspnea	Chronic dyspnea, cough Decrease in FEV_1_ and FEV_1_/FVC ratio	Chronic dyspnea Decrease in FEV_1_ and TLC	Acute dyspnea Decrease in FEV_1_
CT signs	Ground-glass opacities Interlobular septal thickening Pleural effusions Volume loss	***“Obstructive phenotype”*** Bronchiectasis Bronchial thickening Expiratory air-trapping	***“Restrictive phenotype”*** Bronchiectasis Peripheral consolidations or ground-glass opacity Subpleural thickening Architectural distortion Upper lobe predominance Volume loss	Inter-/intralobular septal thickening Peripheral ground-glass opacity and consolidations

AFOP: acute fibrinous organizing pneumonia; BOS: bronchiolitis obliterans syndrome; FEV_1_: forced expiratory volume in 1 s; FVC: forced vital capacity; RAS: restrictive allograft syndrome; TLC: total lung capacity

### Acute allograft rejection

Acute allograft rejection occurs mostly during the first year following transplantation in almost 30% of recipients and may occur as repetitive episodes [1]. Prompt diagnosis and management are necessary because early and repeated exacerbations of acute rejection may lead to CLAD. Many attempts have been made to identify specific signs of acute rejection on CT, but, considered individually, none seem accurate nor have a good predictive value for acute rejection and the degree of severity [12]. Nevertheless, recognition and reporting of CT signs of potential acute allograft rejection are warranted to guide transbronchial biopsy for histopathological analysis.

**Fig. 7:**
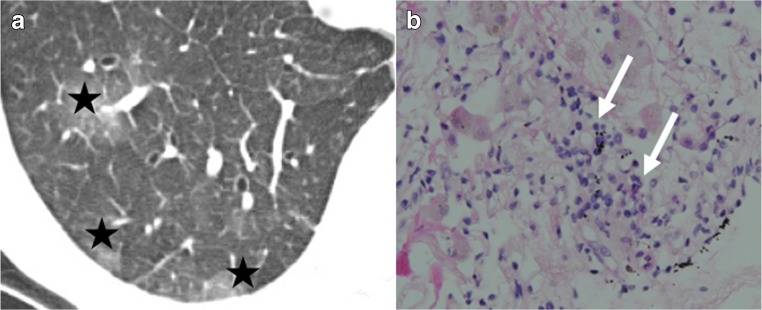
Acute rejection A1. Ground-glass opacities (*stars*) and interlobular thickening (*arrowheads*) (**a**) in minimal acute rejection, characterized by sparse perivascular mononuclear infiltrates (*arrows*) (**b**)

**Fig. 8:**
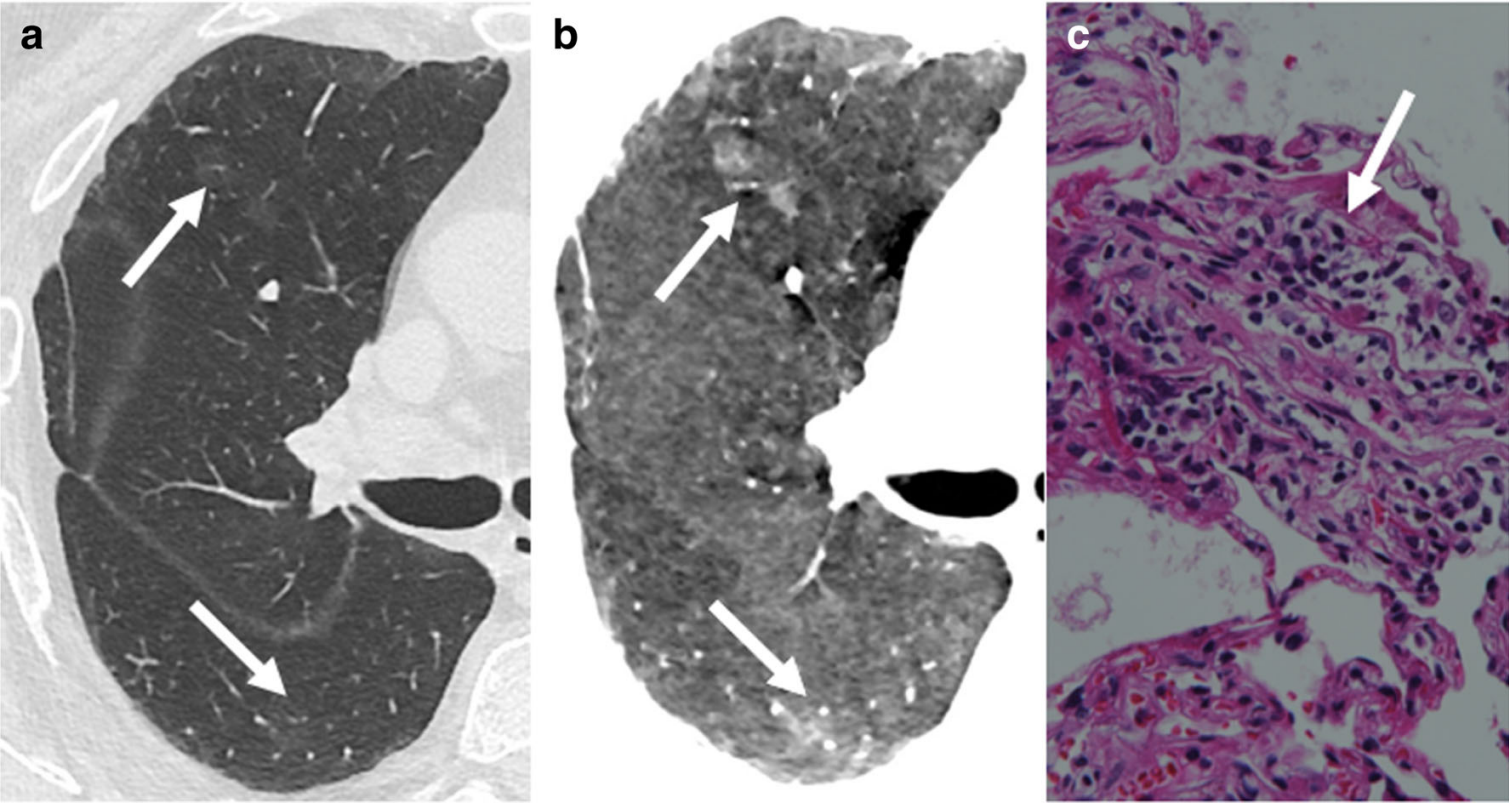
Acute rejection A2. Subtle ground-glass opacities (*arrows* in **a**) better detected with minimal intensity projection (*arrows* in **b**) in mild acute rejection, with more frequent perivascular mononuclear infiltrates and endothelialitis (*arrow* in **c**)

**Fig. 9:**
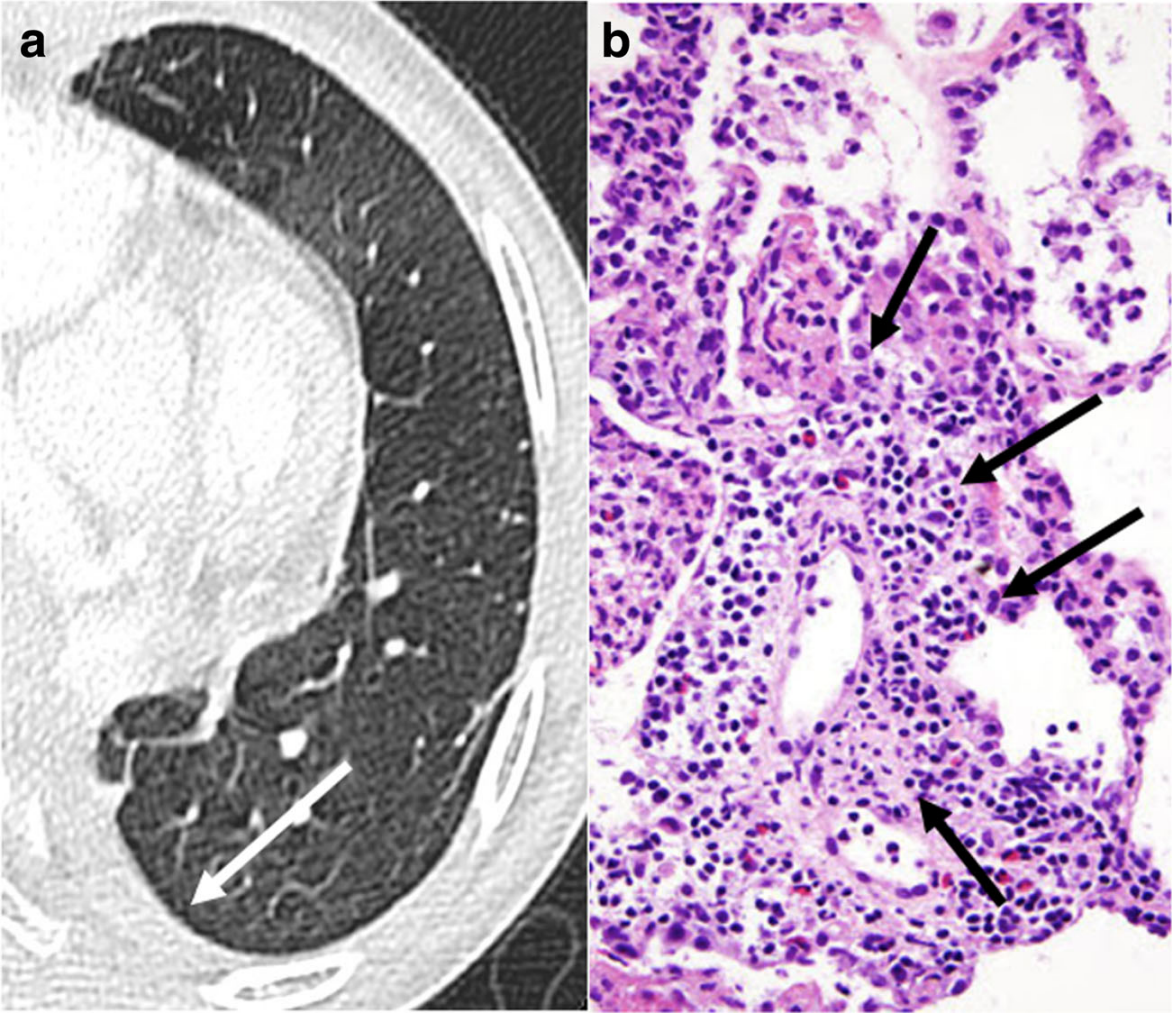
Acute rejection A3. New pleural effusion (*arrow*) (**a**) as the only finding in moderate acute rejection, histologically defined as dense perivascular mononuclear infiltrates with peribronchiolar and airspaces extension (*arrows*) (**b**)

Typical CT signs are ground-glass opacities, pleural effusions, lung volume loss, and interlobular septal thickening, associated with graduate perivascular and interstitial mononuclear cells infiltration depending on the grade (from minimal perivascular mononuclear infiltrates [grade A1] to severe infiltrates [grade A4]) on histopathology examination (Figs. 7, 8, and 9) [7, 13].

### CLAD

The prevalence of CLAD 5 years after transplantation is 40–50% of patients [1, 14]. CLAD has two distinct phenotypes: obstructive, namely, bronchiolitis obliterans syndrome (BOS) and restrictive, namely, restrictive allograft syndrome (RAS) [15, 16]. CT scans along with lung functions may help in distinguishing between subtypes.

**Fig. 10:**
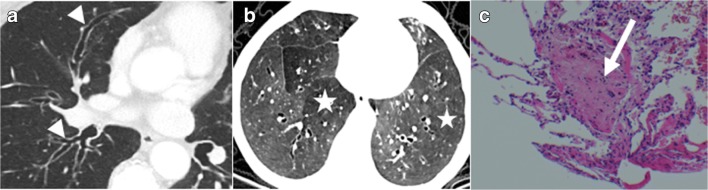
Bronchiolitis obliterans syndrome (BOS). Chronic dyspnea with obstructive pattern at functional tests. Bronchiectases, bronchial thickening (*arrowheads*) (**a**) and mosaic perfusion on minimal intensity projection reconstruction (radiolucent areas; *stars*) (**b**) in constrictive bronchiolitis, characterized by submucosa fibrosis with mononuclear infiltrates of the small airways (*arrow*) (**c**)

**Fig. 11:**
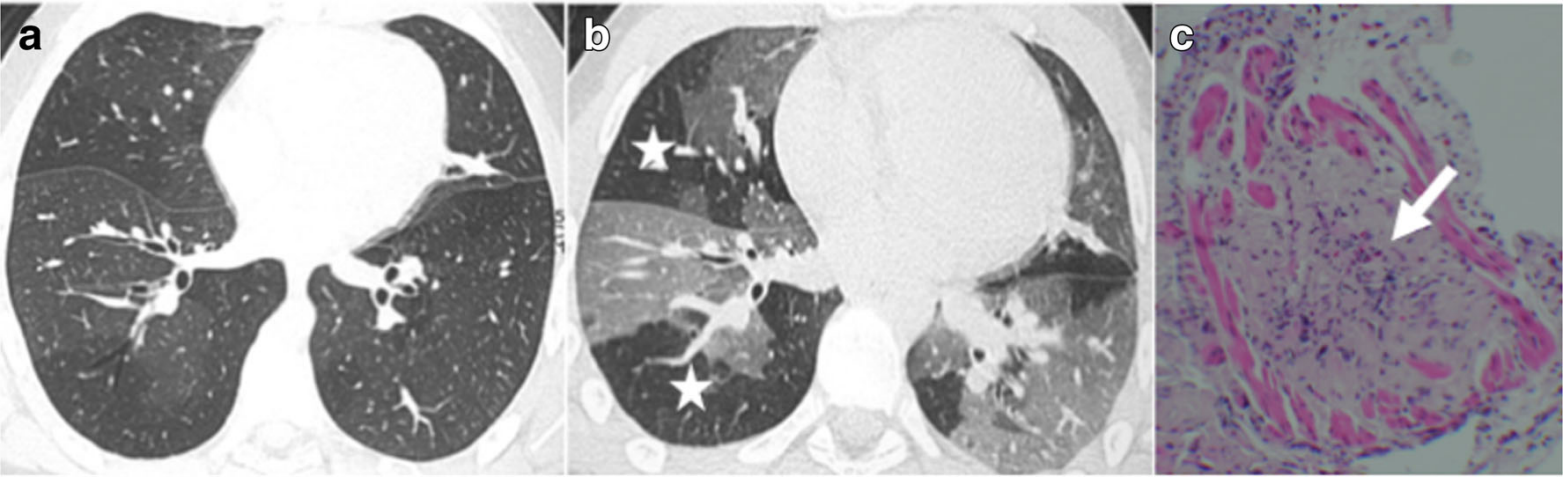
BOS. Chronic dyspnea and cough. Normal parenchyma on inspiration acquisition (**a**) followed by air-trapping on expiration acquisition (*stars*) (**b**) in constrictive bronchiolitis, characterized by submucosa fibrosis (*arrow*) (**c**)

BOS is a constrictive bronchiolitis [13]. CT signs of BOS are bronchiectasis, bronchial thickening, and air-trapping on expiratory acquisition (Figs. 10 and 11) [4, 17–19].

**Fig. 12:**
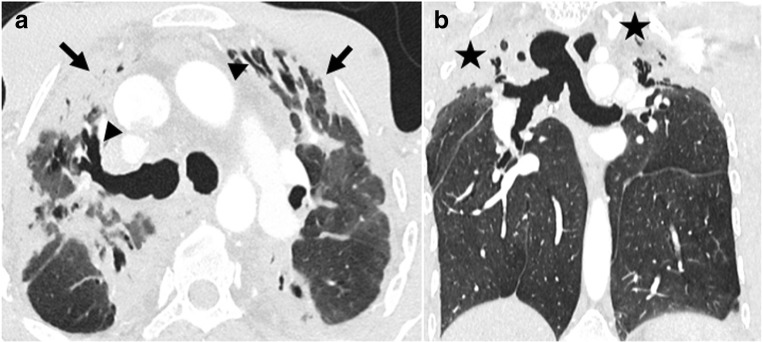
Restrictive allograft syndrome (RAS). Dyspnea and irreversible decline in forced expiratory volume (FEV) and total lung capacity (TLC). Peripheral condensations and subpleural thickening (*arrows*), bronchiectasis (*arrowheads*) (**a**) and volume loss with upper lobe predominance (*stars*) (**b**). RAS was confirmed by biopsy

Conversely, RAS is a pleuroparenchymal fibroelastosis on histopathology [20], with a more fibrotic pattern on CT and poorer survival. CT signs of RAS are peripheral consolidations or central or peripheral ground-glass opacities, septal or non-septal lines, subpleural thickening, bronchiectasis, architectural distortion, and volume loss with an upper lobe predominance (Fig. 12) [21].

**Fig. 13:**
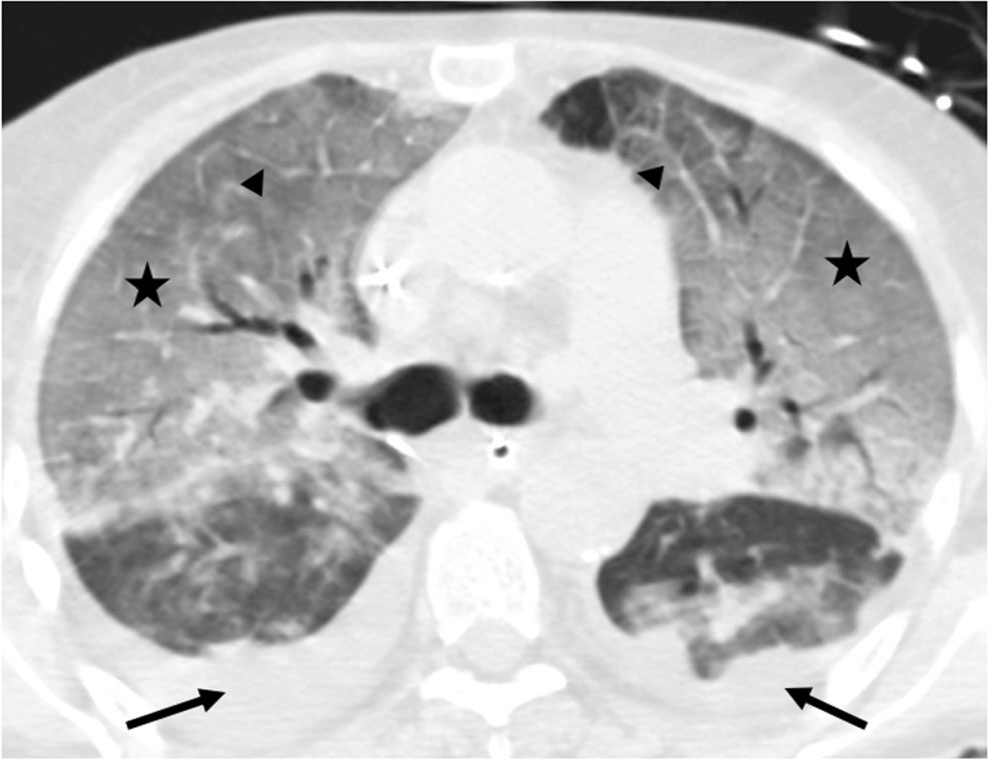
Acute fibrinoid allograft pneumonia (AFOP). Acute shortness of breath. Intralobular septal thickening and extensive ground-glass opacities (*stars*), interlobular thickening (*arrowheads*), and pleural effusions (*arrows*), confirmed by histology

Some authors have advocated acute fibrinoid organizing pneumonia (AFOP) as a third potential form of chronic allograft dysfunction, with decline of lung functions as for CLAD but with distinct histopathology and imaging findings [22]. Inter- and intralobular septal thickening, extensive ground-glass infiltration, and peripheral consolidations have been described and are consistent with a distinct histopathology entity, either post-infectious or as a distinct form of chronic allograft dysfunction (Fig. 13) [22].

## Infections

Lung-transplanted patients are at particular risk of allograft infections and radiologists should be aware of the epidemiology of pneumonias according to their time of onset. Table 3 summarizes different patterns according to the type of infection.

**Table 3:** Specific CT pattern with various infections

Infectious diseases	Bacterial germs	Viral germs	Angioinvasive aspergillosis
Time of occurrence	0–4 weeks	4 weeks to 6 months	4–18 months
Clinical keys	Productive cough and high-grade fever/sepsis Bronchoalveolar lavage Sputum culture	Productive cough and high-grade fever	General alteration and productive cough Bronchoalveolar lavage: branching filaments
CT signs	Lobar or patchy consolidations Air bronchogram Branching centrilobular nodules Cavitation Pleural effusion	Ground-glass nodules Tree-in-bud nodules Peribronchovascular thickening Interlobular septal thickening	Vascular-centered nodules Ground-glass “halo sign” Subpleural wedged ground-glass opacity or dense consolidation

The aim of prompt recognition of allograft infection is to reduce immediate morbidity related to symptomatic disease thanks to prompt targeted therapy and to prevent failure of anastomotic healing and long-term chronic allograft dysfunction [5].

**Fig. 14:**
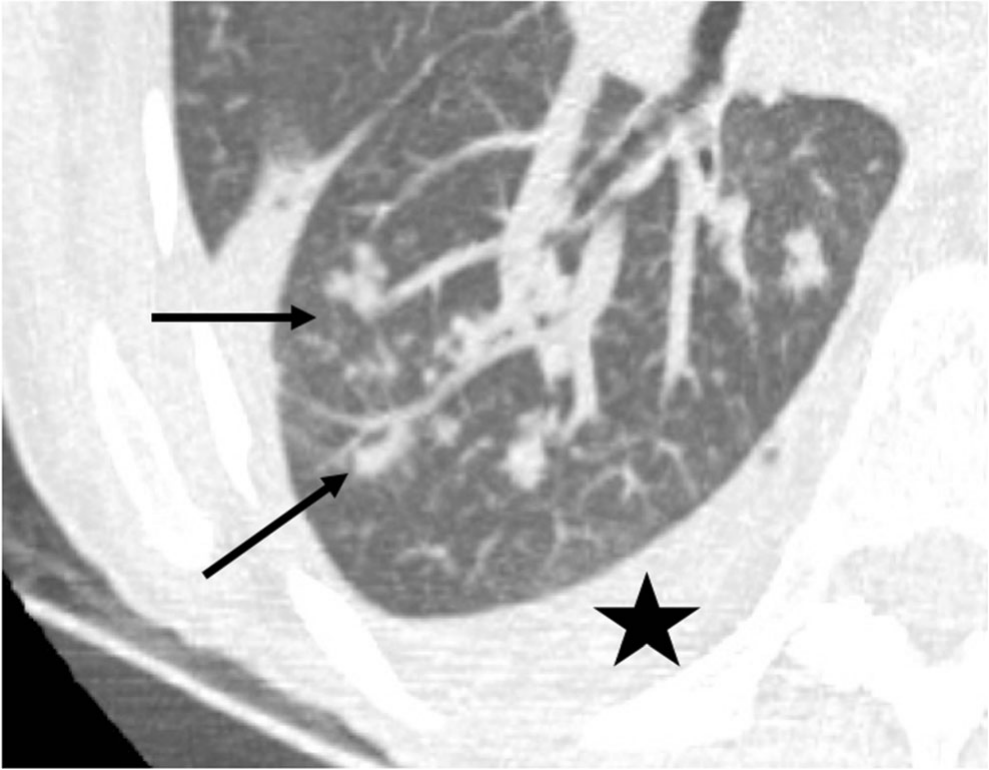
*Pseudomonas aeruginosa*. Productive cough and high-grade fever. Dense tree-in-bud centrilobular nodules on maximal intensity projection reconstruction (*arrows*) and pleural effusion (*star*). *Pseudomonas aeruginosa* was confirmed by sputum culture

**Fig. 15:**
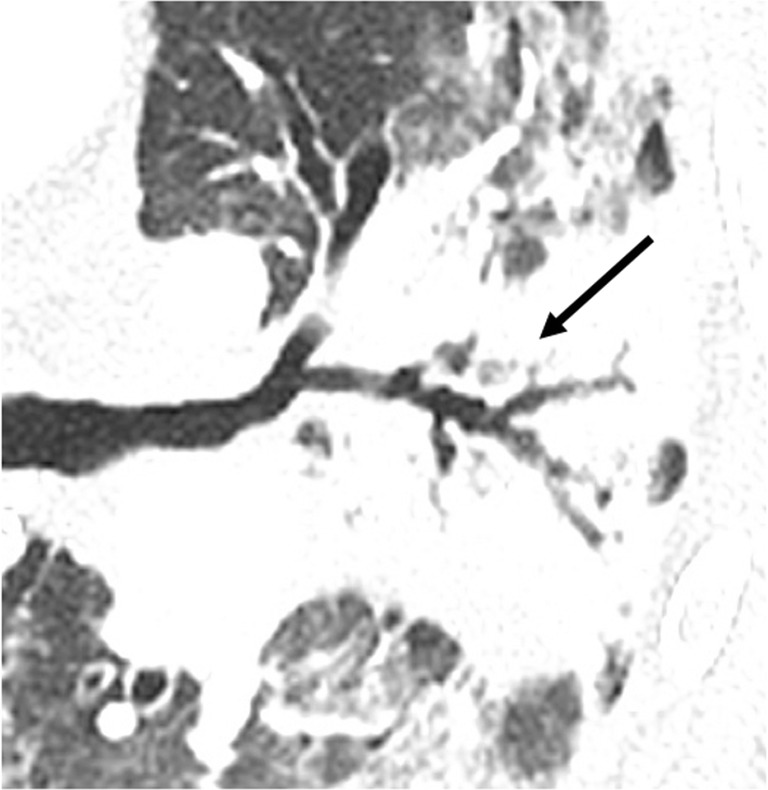
*Staphylococcus epidermidis*. Sepsis. Consolidation with air bronchogram (*arrow*)

**Fig. 16:**
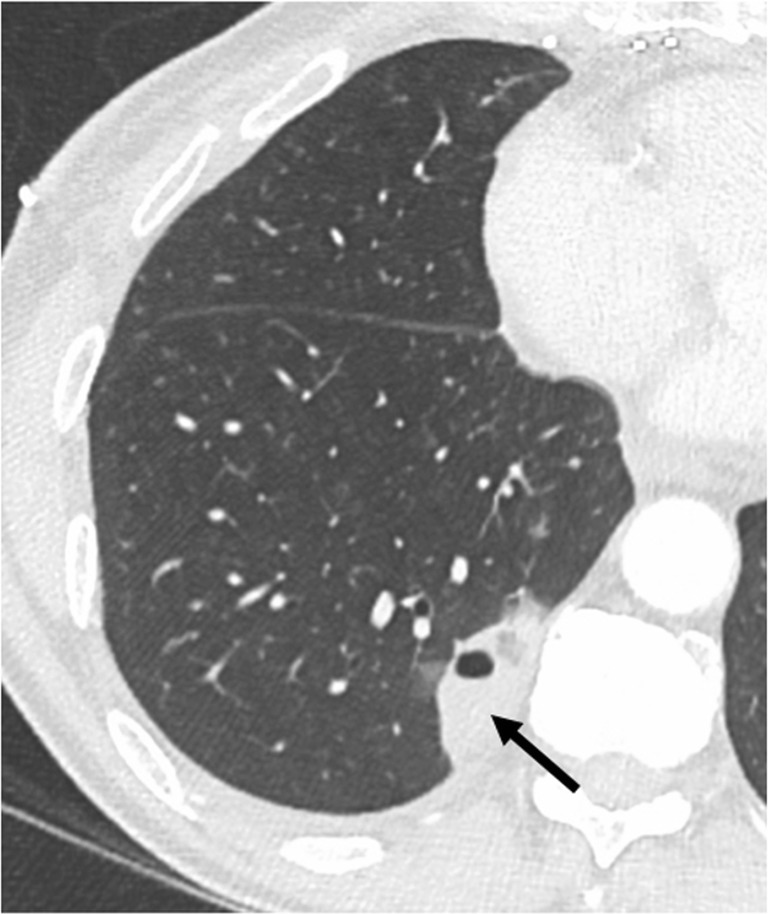
Lung abscess. Fever of unknown origin and asthenia. Consolidation with air–fluid level (*arrow*) suggestive of lung abscess. Methicillin-susceptible *S. aureus* (MSSA) infection was confirmed by bronchoalveolar lavage

The first post-operative month is mostly associated with bacterial and fungal pathogens [6, 23]. Bacterial infections are the most common. CT signs of bacterial infections include extensive consolidations with air bronchogram, disseminated patchy consolidations, branching centrilobular nodules (Figs. 14 and 15), or cavitation or abscess (Fig. 16).

**Fig. 17:**
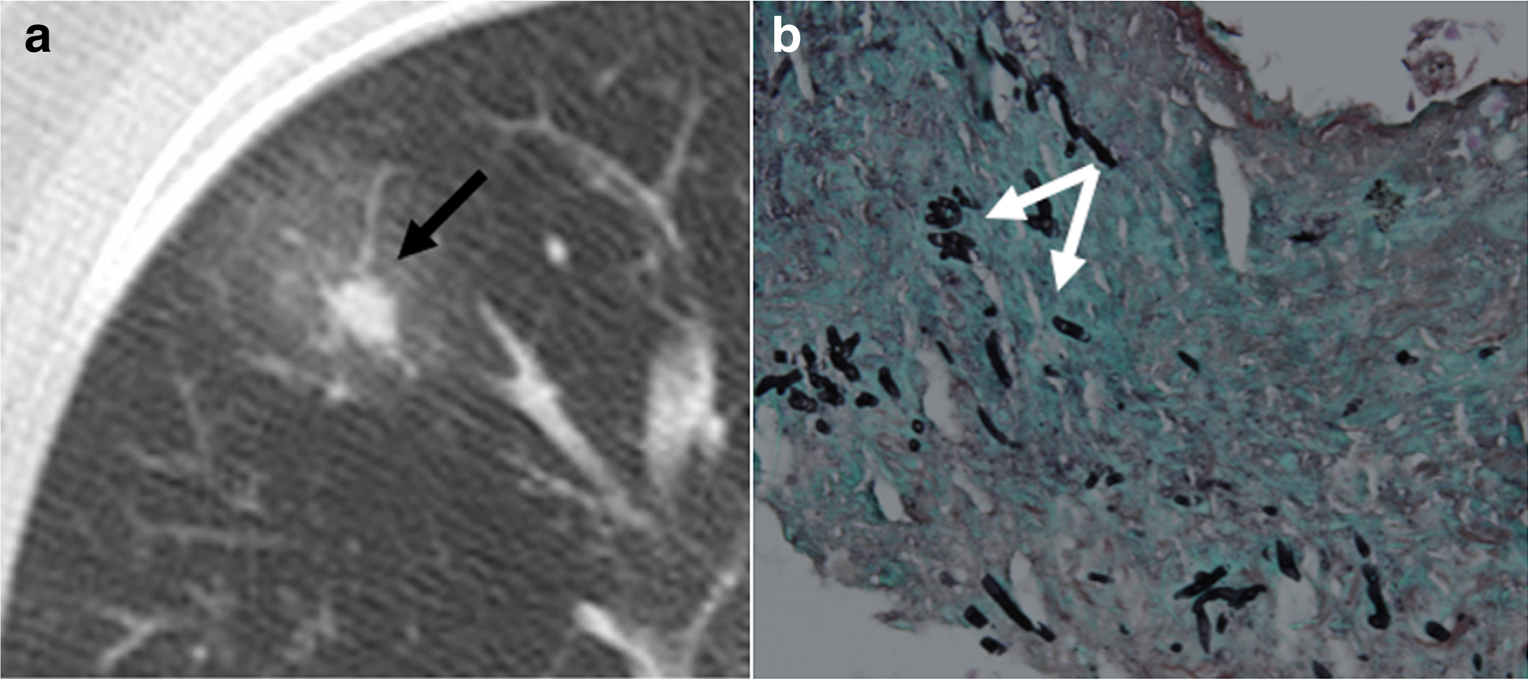
Angioinvasive *Aspergillus fumigatus*. General alteration and productive cough. Solid nodule with peripheral ground-glass opacities termed as the “halo sign” (*arrow*) (**a**). Pathology showed branching filaments (*arrows*), consistent with angioinvasive aspergillosis (**b**)

CT signs of angioinvasive aspergillosis are isolated or multiple vessel centered nodular consolidations with peripheral ground-glass “halo sign” consistent with hemorrhagic infarcts (Fig. 17) [24].

**Fig. 18:**
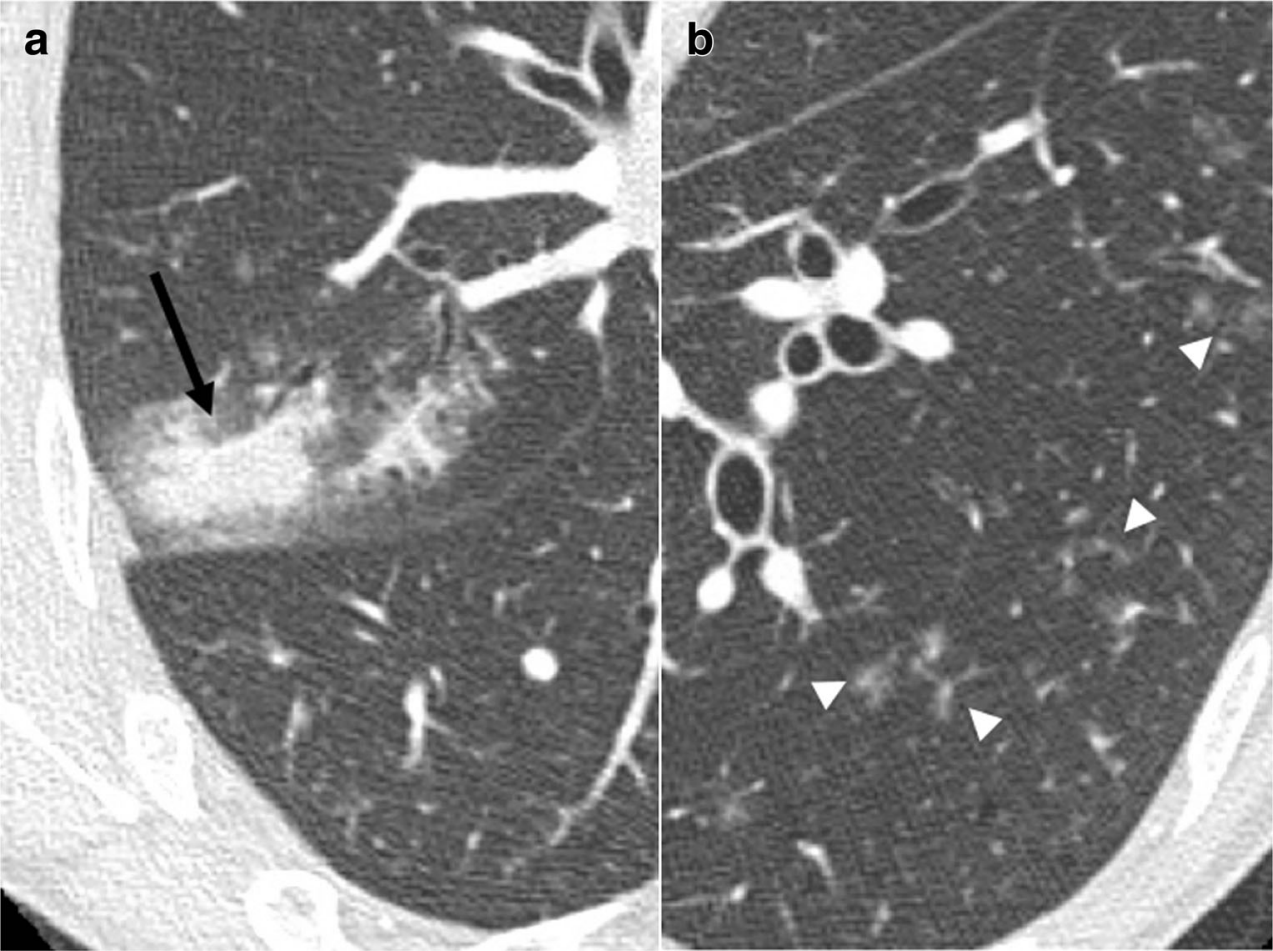
Parainfluenzae pneumonia. Sepsis. Segmental consolidation of the right upper lobe (*arrow*) (**a**) and left basal ground-glass centrilobular micronodulation (*arrowheads*) (**b**)

From the second to sixth months, long-term immunosuppression of T-cells is responsible for viral pneumonias. CT signs are ground-glass opacities or tree-in-bud nodules (Fig. 18). Fungal and parasitic opportunistic infections can also occur, either from reactivation of latent germs, in particular from cytomegalovirus (CMV), or by community-acquired or nosocomial transmission [6]. CT signs are also ground-glass opacities, tree-in-bud nodules, or an interstitial pattern with peribronchovascular and septal thickening [7].

After six months, immunosuppression is usually reduced with less opportunistic infections or reactivation of latent pathogens. The most commonly encountered pathogens are community-acquired viral and bacterial, or reactivation of latent *Mycobacterium tuberculosis* and other mycobacteriae [6, 7]. Individual CT signs are of limited accuracy to distinguish between germs [24]; yet, recognition of some specific patterns of involvement, such as consolidations with air bronchogram, tree-in-bud, or nodules in the context of fever, may favor infection over an alloimmune reaction.

## Malignancy

**Fig. 19:**
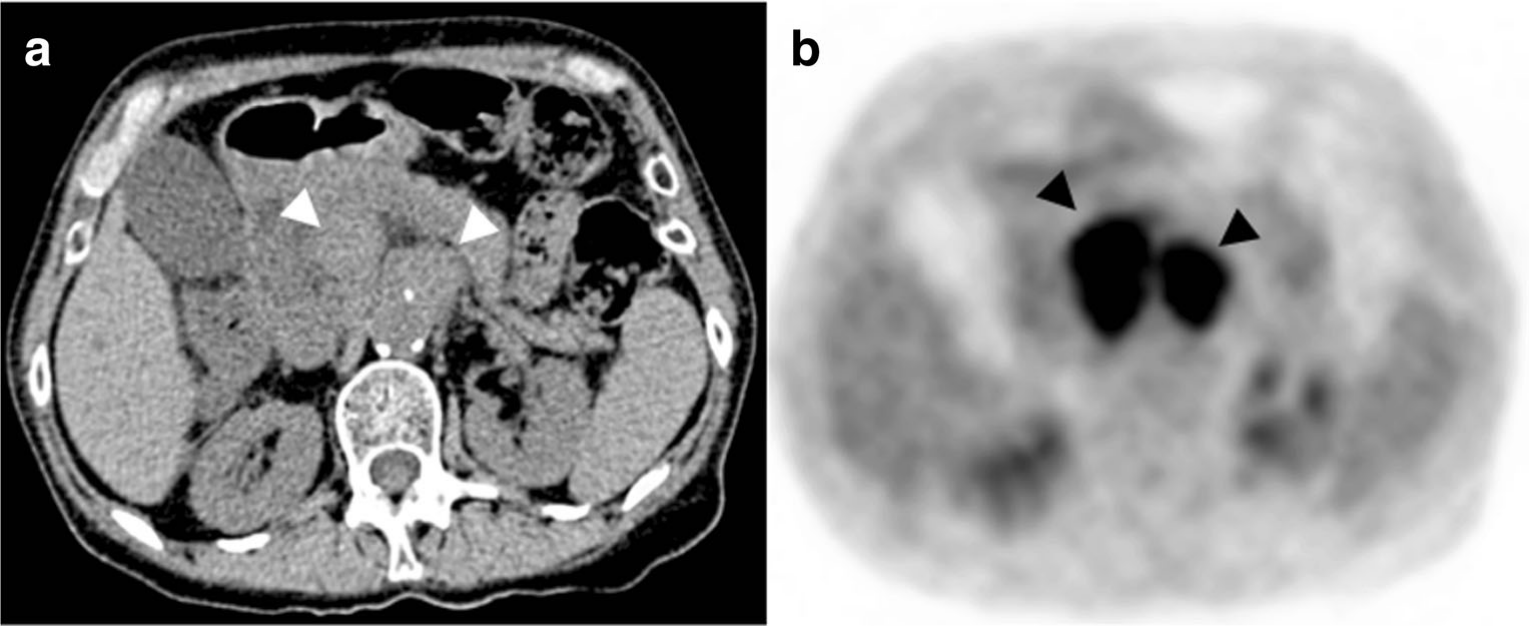
Post-transplantation lymphoproliferative disease (PTLD). Weight loss and general alteration. Voluminous retroperitoneal lymphadenopathies on non-enhanced abdominal CT (*arrowheads*) (**a**) with increased metabolism on FDG-PET CT scanning (*arrowheads*) (**b**)

Finally, malignancy remains an important cause of long-term mortality, occurring in almost 25% of patients 5 years after lung transplantation [1, 2]. Malignancy may be confined to the lung allograft or involve distant organs. Post-transplantation lymphoproliferative disease (PTLD) includes a spectrum from lymphoid proliferation to monoclonal lymphoma, affecting 1.8–20% of lung-transplanted patients, with am association with Epstein–Barr virus infection [6, 25]. The manifestation of PTLD includes an isolated nodule or mass of the lung allograft, disseminated micronodules with an interstitial topography, or mediastinal or abdominal lymphadenopathies (Fig. 19) [25].

**Fig. 20:**
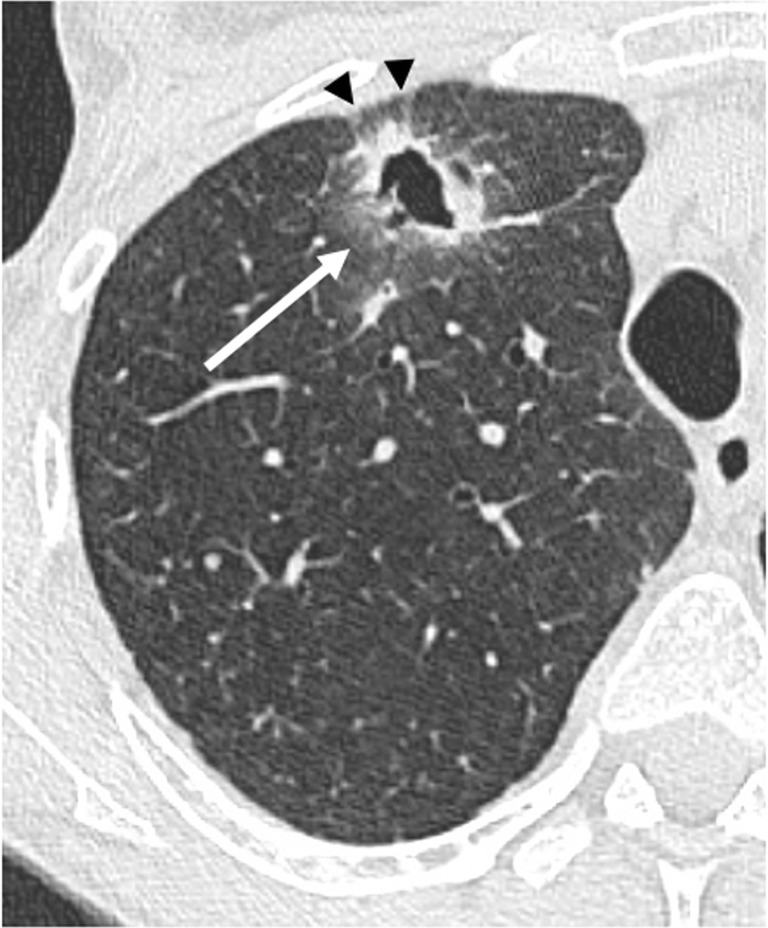
Primary epidermoid lung carcinoma. Asymptomatic, cavitated nodule on follow-up chest X-ray. Cavitated nodule with spiculated margins (*arrow*) and pleural traction (*arrowheads*)

Skin malignancies are also predominant, including melanoma, reaching nearly 20% at 10 years. Other cancers could also occur, such as primary epidermoid lung carcinoma (Fig. 20).

## Conclusion

Chest computed tomography (CT) scanning is an important tool in the follow-up of lung transplantation, allowing the diagnosis of pathologies depending on their time of onset in order to improve patient management.

## Abbreviations

AFOP: Acute fibrinous organizing pneumonia
BOS: Bronchiolitis obliterans syndrome
CLAD: Chronic lung allograft dysfunction
CMV: Cytomegalovirus
CT: Computed tomography
PGD: Primary graft dysfunction
PTLD: Post-transplantation lymphoproliferative disease
RAS: Restrictive allograft syndrome
